# Critical aspects to enable viable solar-driven evaporative technologies for water treatment

**DOI:** 10.1038/s41467-022-33533-0

**Published:** 2022-10-03

**Authors:** Eliodoro Chiavazzo

**Affiliations:** 1 Department of Energy, Politecnico di Torino, Corso Duca degli Abruzzi 24, 10129 Torino, Italy; 2 Clean Water Center, Corso Duca degli Abruzzi 24, Torino, 10129 Italy

**Keywords:** Mechanical engineering, Nanoscale materials, Applied physics, Thermodynamics

## Abstract

While passive solar-driven evaporative systems promise higher economic and environmental sustainability in water treatment, many challenges remain for their effective adoption. Here, the author identifies three main pillars and corresponding issues which future research should focus on to bring these technologies to the next maturity level.

## Teaser

Recent studies in passive solar-driven evaporative technologies have introduced a plethora of new materials and devices which promise higher economic and environmental sustainability in water treatment. However, many challenges remain for the effective adoption of such technologies. Here, we identify three main pillars and the corresponding issues which future research activities should focus on to bring the proposed solutions to the next maturity level. Specifically, our analysis focuses on standards for comparing productivity, strategies to overcome the single stage limit, scalability and robustness.

Water scarcity is a major issue affecting billions of people all over the world^1^. As a result, the sixth Sustainable Development Goal (SDG), addressing access to clean water, plays a critical role further exacerbated by climate change, urbanization and population growth^2^. Meeting such a goal at a large scale is a multifaceted issue and requires disparate actions at different levels including new policy strategies and better infrastructures. However, it seems inconceivable that water scarcity can be fully alleviated without a significant technological advancement towards efficient and extensive exploitation of seawater, the largest water source on Earth. Treatments are required for effectively separating fresh water from salt or other contaminants, and the adoption of renewables (specifically solar energy) represents an obvious and possibly sustainable option to drive desalination and other water treatments in general.

In recent years, starting from the seminal work of Chen and collaborators^3^, the concept of solar steam generation by heat localization has been extensively investigated. Many photothermal functional materials have been synthetized and tested for processing salty (or contaminated) water. While a general overview of those solutions has been comprehensively reported elsewhere^4–7^, the main scope of this Comment is to critically analyze and put into perspective some outstanding issues that impact the effective adoption of such technologies.

The materials and devices of interest here typically embody a number of features, namely: (i) *all-in-one* solutions where several functions (e.g. solar energy collection, water wicking, flow regulation, photothermal conversion, self-floating) are present in a single system; (ii) over 80% overall first-law efficiency in the sun-to-steam process (thanks to reduced heat losses to the environment as compared to solutions based on bulk water heating); (iii) generally passive systems which operate without moving parts and without the need of additional ancillary mechanisms, similarly to the well-known solar stills. As such, passive approaches are potentially characterized by low capital and operating costs, making them attractive especially in isolated and impoverished areas. As a side note to the latter point, we stress that the passive nature of such systems has limitations: Even if fresh water can be passively generated *in loco*, water supply to the users will anyway require pumps or alike regardless of the adopted system.

Focusing on seawater desalination to fix ideas, fresh water production is known to be an energy intensive process. Thus water and energy access are intimately intertwined issues to address in modern society (water-energy nexus). Further, a direct comparison of different technologies in terms of the energetic cost is nontrivial, especially for drivers of disparate nature. An effective approach is based on the equivalent work, where the energy input is weighted by its capacity to be converted into electricity: According to^8^, in large plant installations, state-of-the-art active solutions present an energetic cost in the range of 3–6 [kWh m^−3^] equivalent work.

In contrast, even assuming 100% efficiency of the photothermal process, any passive technology performing a single distillation cycle (i.e. vaporization followed by condensation with heat rejected into the environment) requires the enthalpy of water vaporization^9^ amounting to over 640 [kWh m^−3^] of thermal energy. While the latter figure might be further converted into equivalent work, the energy cost of such technologies remains orders of magnitude higher than the one realized in large active desalination plants. On one hand, if such large energy input was to come from solar radiation alone, this would be reflected in large installation areas and capital costs thus possibly leading to a niche use in impoverished areas if we only focus on water desalination. However, in other applications, such passive technology may make an important impact even in developed countries. For instance, wastewater treatment by evaporation ponds could benefit by photothermal enhancement at a sustainable cost increase.

Consolidating on former analysis^10^, below we identify and critically analyze important aspects that we believe the scientific community should further consider for the field to move towards more comprehensive sustainability and technological maturity.

## Comparing productivity

One of the most important and reported figures of merit is the specific fresh water (or vapor) productivity per unit of area and unit of time (often expressed in the physical units of [liter h^−1^ m^−2^] or [kg h^−1^ m^−2^]). Major aspects affecting productivity include steam generation only as opposed to liquid water, and the use of optical concentration. In desalination, liquid water collection is more important. Operating conditions with optical concentration exceeding unity (i.e. energy fluxes higher than 1 [kW m^−2^]), have to be carefully evaluated. In those cases the economic and technical advantages of passive solutions do not appear obvious^11^ and require a *case-by-case* study. Even with energy fluxes lower than 1 [kW m^−2^] (no optical concentration), a meaningful comparison of solar-driven evaporative systems in terms of water productivity requires a standardization of the operating conditions as well as an unambiguous definition of the figure of merit itself and the corresponding normalization parameters.

Operating conditions can differ as some systems have been studied at the lab scale where light is generated under a solar simulator and other devices undergo an *in-field* or *in-sea* characterization^3,12^. Therefore, a direct comparison of data obtained under such diverse operating conditions is questionable. One of the reasons may be linked to the class of the solar simulator used^13^. However, a major challenge remains the exact control of all relevant optical conditions (and energy fluxes) within the enclosed space of a laboratory. It suffices to notice that the optical view factor of the evaporating surface and the solar simulator lamp will be dramatically different as compared to the same surface exposed to the Sun in the sky. Similar considerations apply to the lab walls as compared to buildings, vegetation etc. within a real *in-field* installation. Moreover, in order to properly account for heat convection losses, the air or wind speed and direction is to be measured and reported.

This suggests that well-defined and clear reference testing conditions should be identified and agreed upon in the community with different conditions for various cases (i.e. *in-field* and *in-labs*). We envision the exact definition of three benchmark case studies, each defined by a minimum amount of well-controlled operating conditions as reported in Table 1. In addition to the above benchmark cases, it would also be desirable to define an *unsteady* laboratory benchmark test where the imposed energy flux follows a prescribed time dependent law in order to standardize and characterize transient system behaviors. If accomplished, current technologies could undergo comprehensive round robin studies, whereas future works can claim groundbreaking results by directly conducting experiments under the exact reference conditions set by the community.

**Table 1:** Checklist table of requested operating conditions for comparing different technologies

	Benchmark #1	Benchmark #2	Benchmark #3
Testing modality	In laboratory	In field	In sea
Average energy flux	✓	✓	✓
Solar simulator class	✓	X	X
Ambient air temperature	✓	✓	✓
Ambient relative humidity	✓	✓	✓
Lamp-evaporative surface view factor	✓	X	X
Wall-evaporative surface view-factor(s)	✓	X	X
Wall temperature(s)	✓	X	X
Wind/air average speed	✓	✓	✓
Input water salinity	✓	✓	✓
Input water turbidity and total organic carbon	✓	✓	✓
Target output	Steam/liquid water	Steam/liquid water	Steam/liquid water

Fields with check mark indicate the desirable least amount of critical information to control and report in comparative studies. The remaining fields (with the symbol X) denote unnecessary or irrelevant information.

Recent attempts have been reported where the concept of 3D interfacial evaporators have been suggested over the classical 2D solar evaporators. 3D evaporators introduce a smart design where hydrophilic stalks with high aspect ratio are vertically placed in contact with water at the bottom, whereas communication with air occurs through the lateral and the top surface. Although water vapor is released into air laterally, the figure of merit on productivity is computed adopting the much smaller projected ground area which, for a single cylindrical unit, corresponds to the base area. An astonishing yield exceeding 30 [kg m^−2^ h^−1^] could been thus observed^14^. In addition, such design has attracted interest as it may offer superior fouling control^15^.

Such a choice comes with a few aspects that require care. The specific productivity as defined above is strictly representative of single evaporators, or sparse arrays, as densely-packed 3D evaporators are not expected to linearly scale with the array extension in terms of productivity. Preliminary results on 3D evaporators arrays in more realistic conditions show productivity values in the order of 10 [kg m^−2^ h^−1^] or lower^16^. In real applications, an important target is maximization of fresh water output per unit of surface area occupied by the desalination plant. Hence, more studies are desirable to achieve a more comprehensive and theoretical understanding of the collective behavior of such 3D vertical evaporators with particular focus on development and interference of fluid-dynamic boundary layers of each element in the array.

An even more crucial aspect to be highlighted is that solutions relying on the enhancement of vapor flux due to lateral air convective flows exploit mass transfer phenomena towards non-saturated ambient air. This comes with two points of attention.

First, performance of 2D and 3D evaporators are not fairly comparable as 2D evaporators are driven by the exergy content of sunlight whereas 3D evaporators exploit both exergy content of sunlight and chemical exergy associated to non-saturated ambient air. Accessibility to the latter exergy source might be limited in desalination systems, although it can be exploited in applications based on evaporation only, such as wastewater treatment^15^.

Second, 3D evaporators generate cold water vapor, eventually at near or sub-ambient conditions^17^. The final output of desalination or distillation technologies is requested in the form of liquid water, and evaporation is only one of a two-step distillation process. The colder the water vapor during the first evaporative step, the more challenging the subsequent condensation as it requires an even colder surface capable of taking the latent heat up to reject it to ambient. Hence, a more meaningful comparison of 2D and 3D technologies in terms of specific water productivity can be carried out if both systems deliver liquid water as a final product. In this sense, it remains to be investigated to which extent 3D evaporative systems, if embedded within an evaporation and condensation cycle, can reach superior performance as compared to highly efficient 2D solutions^12,18^. Finally, it would be desirable to introduce volumetric figures of merit for 2D and 3D systems where water productivity per unit of time and occupied volume is also evaluated (in [kg m^−3^ h^−1^]).

## Overcoming the single stage distillation limit

Solutions realizing one complete distillation cycle remain limited by the following thermodynamic limit: 1,47 [liter kWh^−1^] corresponding to a productivity figure of 1,47 [liters m^−2^ h^−1^] when operating with an energy flux of 1 [kW m^−2^]^12^. Even if operating at such thermodynamic limit, without resorting to optical concentration, large-area installations are needed to fulfil just the drinkable water needs of a single person on average, namely 2.5 liters per day^19^. The latter figure though could be significantly higher if other daily water needs are also included. Two main strategies have been proposed to overcome the above productivity limit.

On one hand, it has been recognized that, similarly to large desalination plants, heat management within the passive device is crucial. Here, multiple distillation stages are piled up with the aim of re-using several times the latent heat of condensation while keeping the passive nature of the device^12,20^. The latter approach enabled to observe productivity values exceeding 5,7 [liters m^−2^ h^−1^] under one sun illumination^18^.

On the other hand, some studies have been focusing on materials that may help reducing considerably the water vaporization enthalpy, by postulating that in some materials (e.g. hydrogels) evaporation proceeds according to the water cluster theory: Namely, water is not evaporated as single molecules but rather in small clusters made of a few or dozens of molecules. Since the first observation in 2018^21^, the reduction of water vaporization enthalpy has been mostly hypothesized to indirectly explain the energy balance in a few studies. However, this has still not been confirmed by direct measurements, neither in hydrogel^22^ nor in other metal-based materials^23,24^, thus remaining a debated issue in the scientific community.

To date, it is unclear whether the water cluster evaporation is a thermally driven process or if it requires the light mediation. In the former case, more extensive calorimetric studies are required. Specifically, in addition to measurements based on the first law balance of evaporating materials under non-equilibrium conditions, researchers should also focus on experiments targeting measurements under equilibrium conditions exploiting the Clausius-Clapeyron relationship: Measuring equilibrium water adsorption isotherms and isobars as suggested in^13^ could be helpful, although it might not be fully comprehensive. In fact, recent research avenues seem to suggest that water cluster evaporation (and thus vaporization enthalpy reduction) might be driven directly by light, thus realizing the recently reported *photo-molecular* effect in which photons are believed to be directly responsible for cleaving water molecules clusters off from the liquid-vapor interfaces^25,26^. If further confirmed by the scientific community, this approach may open new exciting and yet unimaginable possibilities in the field. Finally, we observe a lack of fundamental studies (e.g. numerical simulations) capable of enhancing our theoretical understanding at the molecular level on such possible vaporization enthalpy reduction.

## Scalability and robustness

The intrinsic upscaling of lab-scale characterized devices or materials will likely determine whether solar-driven passive technologies for water treatment will reach full maturity in the coming years.

On one hand, solid and consolidated evidence on technological robustness with respect to extensive cyclability and aging is still lacking. When reported, typical testing timescales are limited to weeks^20^ and only a dozen of cycles^27^. Nonetheless, the expected low maintenance over long time periods (on the order of years) is a key aspect of these passive technologies as demonstrated by a recent technoeconomic analysis in^10^. To this end, advances in material science appear promising for realizing cost-effective and durable material with intrinsic anti-clogging properties^28^, however the community will have to keep providing new robust solutions and respective demonstrations with regard to both salt accumulation and organic matter fouling when subject to long-term operation with seawater and or wastewater. It thus appears natural to take advantage of the prior long-standing know-how gained in the field of membranes for nanofiltration^29^. Interestingly, such approaches can be also combined with highly effective passive flow manipulation exploiting the Marangoni effect^20^ and natural convection^30^ to enhance salt rejection.

Although passive technologies may be less subject to internal wear, when left in open environment their function and structural integrity can be affected by a multitude of conditions such as the action of storms, snow, strong winds, crashing waves, sand, pollution debris, and birds (pecking/scratching/guano). For that, examples can be drawn from other surface modification applications that face similar challenges (e.g. de-icing, self-cleaning, solar cells etc.) which usually require mechanical tests such as sanding, knife scratching, and tape-peeling. Those aspects remain, to date, almost untouched.

Finally, while many of the proposed solutions can safely claim modularity, real economic sustainability requires approaches that are intrinsically scalable to large installation areas. To our knowledge, very few examples have been reported in the literature where massive scalability has been experimentally demonstrated on a surface area of >100 m^2^ ^31^. It appears natural and highly desirable that the community starts increasing the number of studies reporting large scale installations (e.g. >10 m^2^) where estimates of capital and operational expenditure (CAPEX and OPEX) figures can be more realistically achieved. In this directions, modeling and validated computational works for scaling predictions are also requested, since large scale field tests are not always a possibility.

## Concluding remarks

Passive solar-driven technologies for water desalination and/or treatment have attracted an increasing attention and a plethora of different materials and devices have been demonstrated. Despite the undisputable advantages of such approaches, important aspects remain poorly addressed in the current literature, thus creating critical roadblocks towards the complete technological maturity of some ingenious solutions recently proposed. In this work, some of the most important open issues and challenges are identified and categorized within three main pillars. Far from being conclusive, we hope that this Comment will motivate useful actions within the broad research community, thus inducing new breakthroughs towards a more comprehensive sustainability.
